# Chirality-selected phase behaviour in ionic polypeptide complexes

**DOI:** 10.1038/ncomms7052

**Published:** 2015-01-14

**Authors:** Sarah L. Perry, Lorraine Leon, Kyle Q. Hoffmann, Matthew J. Kade, Dimitrios Priftis, Katie A. Black, Derek Wong, Ryan A. Klein, Charles F. Pierce, Khatcher O. Margossian, Jonathan K. Whitmer, Jian Qin, Juan J. de Pablo, Matthew Tirrell

**Affiliations:** 1 Institute for Molecular Engineering, University of Chicago, Chicago, Illinois 60637, USA; 2 Argonne National Laboratory, Argonne, Illinois 60439, USA; 3 Department of Chemical Engineering, University of Wisconsin, Madison, Wisconsin 53706, USA; 4 Department of Bioengineering, University of California at Berkeley, Berkeley, California 94720, USA

## Abstract

Polyelectrolyte complexes present new opportunities for self-assembled soft matter. Factors determining whether the phase of the complex is solid or liquid remain unclear. Ionic polypeptides enable examination of the effects of stereochemistry on complex formation. Here we demonstrate that chirality determines the state of polyelectrolyte complexes, formed from mixing dilute solutions of oppositely charged polypeptides, via a combination of electrostatic and hydrogen-bonding interactions. Fluid complexes occur when at least one of the polypeptides in the mixture is racemic, which disrupts backbone hydrogen-bonding networks. Pairs of purely chiral polypeptides, of any sense, form compact, fibrillar solids with a β-sheet structure. Analogous behaviour occurs in micelles formed from polypeptide block copolymers with polyethylene oxide, where assembly into aggregates with either solid or fluid cores, and eventually into ordered phases at high concentrations, is possible. Chirality is an exploitable tool for manipulating material properties in polyelectrolyte complexation.

## Abstract (web summary)

Complexes that form between oppositely charged polyelectrolytes may be solid or liquid. Here, Perry *et al.* show that chirality in polypeptides can determine the state of those complexes based on a propensity for hydrogen-bond formation.

## Graphical abstract

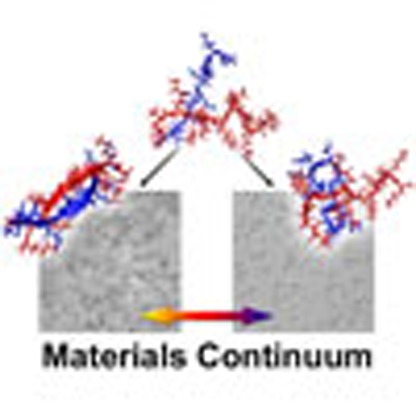

Polyelectrolyte complexation has a long history of utility in encapsulation applications^1^,^2^, produces compartmentalization that is implicated in development of certain biological assemblies^3^,^4^,^5^,^6^,^7^,^8^,^9^,^10^,^11^,^12^ and in origin of life scenarios^13^,^14^, and is accelerating new synthetic materials applications from surface coatings to self-assembled structures^3^,^4^,^5^,^6^,^7^,^8^,^9^,^10^,^11^,^12^,^15^. It is an entropically driven process, where the initial electrostatic attraction between oppositely charged polyelectrolytes is followed by the release of small, bound counter-ions and the restructuring of water molecules^4^,^16^,^17^. Studies on understanding and controlling polyelectrolyte complexation and self-assembly have focused almost exclusively on charge-driven phenomena, such as charge density, pH and ionic strength^4^,^5^,^18^,^19^,^20^,^21^,^22^,^23^. The resulting complexes can form either solid precipitates or liquid complex coacervates^4^. Wang and Schlenhoff^24^ give an excellent account of the progression from precipitate to coacervate to solution in a particular system, but the underlying causes and general understanding of phase selection between solid and fluid state complexes are obscure.

While precipitates result in dense, solid, hydrated materials, fluid coacervate phases retain significant amounts of water (up to 90% in some cases)^18^,^19^, display viscoelastic properties^16^,^22^,^25^,^26^ and have very low surface tension with water^20^,^23^ Complexation can be coupled with molecular design, linking polyelectrolyte domains to hydrophilic, neutral polymer blocks to stabilize microphase separation and drive the assembly of ordered phases associated with traditional block copolymers^3^,^4^,^5^,^6^,^8^,^10^,^12^,^27^,^28^.

A delicate balance of forces determines whether complexation yields a liquid or a solid. This process occurs cooperatively, with the initial electrostatic interaction between the oppositely charged segments of two polymer chains nucleating phase formation^17^. The strength of these interactions can be mediated by charge screening and by parameters such as the acidity or basicity of the charged groups and the density of charges along the polymer^4^,^23^. Higher polyelectrolyte charge density provides an increased stability against salt-induced dissolution of the complexes and enables phase separation with shorter polyelectrolyte chains^18^,^22^,^23^. Higher charge density and strongly charged polyelectrolytes tend to favour solid precipitates over liquid coacervates^4^,^16^. The strength of electrostatic interaction appears to be the one recognized determinant of phase selection.

Various polyelectrolytes have been used in studies of complexation, ranging from charged particles and micelles^29^,^30^ to proteins^4^,^5^,^6^, biologically derived polymers such as gelatin, chitosan and heparin^5^,^31^,^32^, synthetic polymers such as poly(acrylic acid) and poly(allylamine)^3^,^5^,^6^,^21^,^22^ and biomimetic polymers such as polypeptides^5^,^6^,^8^,^12^,^14^,^17^,^19^,^20^. Ionic polypeptides are distinctive owing to the chirality of the monomers and the resulting potential to form secondary structures based on hydrogen bonding. While repulsion among like-charged groups favours the random coil structure of individual polypeptide chains, both α-helical and β-sheet structures can form on charge neutralization or screening^33^,^34^. However, the preponderance of studies on the complexation of polypeptides have used at least one polyelectrolyte lacking the capacity to form secondary structure, such as achiral polymers, polysaccharides or α/β-random co-polypeptides^5^,^6^,^8^,^12^,^16^,^17^,^19^,^20^,^32^,^35^. The effect of the charged side chains in complexation has been well studied in a variety of systems^4^,^5^,^6^,^8^,^12^,^17^,^19^,^20^; however, the effect of backbone chirality on phase selection and materials design in polyelectrolyte complexes has not been tested.

In a broader sense, the stereochemistry of the polymer backbone can have a direct influence on the formation of semicrystalline or amorphous domains. However, these interactions are driven largely by stereoselective van der Waals interactions^36^. Here we examine the chirality effects during polyelectrolyte complexation, taking advantage of not only electrostatic and stereospecific van der Waals forces, but also hydrogen-bonding interactions between polypeptide chains. This diverse set of orthogonal interactions enables the independent tuning of material properties without altering the chemical identity and demonstrates that chirality presents unique opportunities for the manipulation of physical properties in material systems built on polyelectrolyte complexation.

## Results

### Characterization of chirality on bulk polyelectrolyte complexes

**Figure 1:**
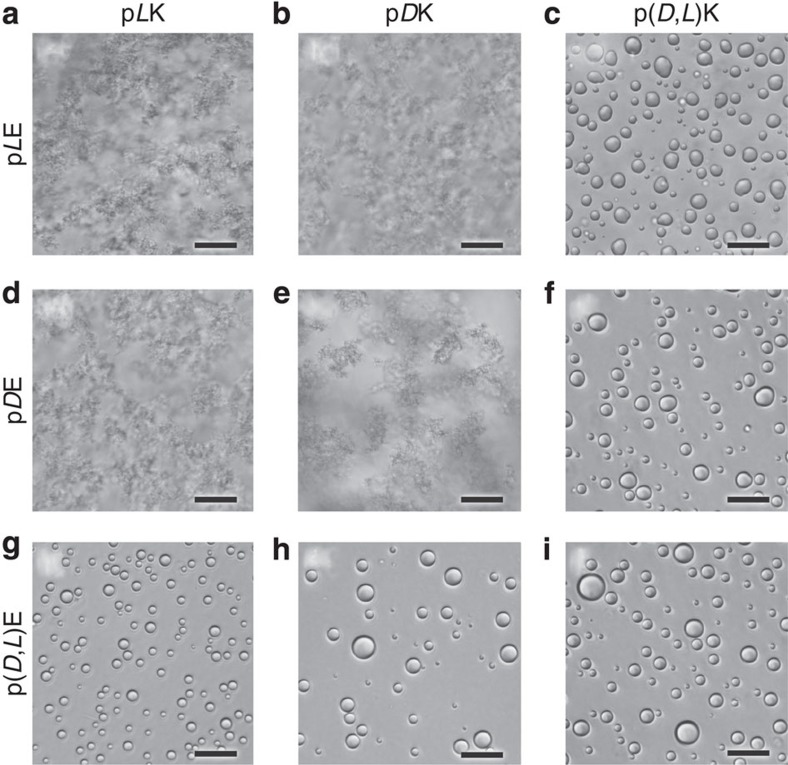
Optical micrographs of polyelectrolyte complexes. Bright-field optical micrographs showing the liquid coacervates or solid precipitates resulting from the stoichiometric electrostatic complexation of *L*, *D*, or racemic (*D*,*L*) poly(lysine) with *L*, *D* or racemic (*D*,*L*) poly(glutamic acid) at a total residue concentration of 6 and 100 mM NaCl. Complexes are formed from (**a**) p*L*K+p*L*E, (**b**) p*D*K+p*L*E, (**c**) p(*D*,*L*)K+p*L*E, (**d**) p*L*K+p*D*E, (**e**) p*D*K+p*D*E, (**f**) p(*D*,*L*)K+p*D*E, (**g**) p*L*K+p(*D*,*L*)E, (**h**) p*D*K+p(*D*,*L*)E, (**i**) p(*D*,*L*)K+p(*D*,*L*)E. Liquid coacervate droplets are only observed during complexation involving a racemic polymer. Scale bars, 25 μm.

We present data here on the complexation of poly(lysine) (pK, using the single-letter abbreviation for amino acids,) with poly(glutamic acid) (pE) while systematically varying the polymer chirality (homochiral; p*L*K, p*D*K, p*L*E and p*D*E and racemic; p(*D*,*L*)K and p(*D*,*L*)E, see Supplementary Table 1 and Supplementary Figs 1 and 2 for details on characterization). Throughout this article, pK stands for polylysine and pE stands for polyglutamic acid; *D* and *L* indicate chirality, with *D*,*L* used to designate racemic polymers. Complexes were formed by mixing stoichiometric amounts of polyanion and polycation (that is, charge-matched conditions), in dilute solution in the presence of varying amounts of NaCl. Visual identification of liquid coacervates or solid precipitates was made using optical microscopy (Leica DMI6000 B). Liquid coacervates appear as small, spherical fluid droplets, while precipitates form amorphous solid clusters. Liquid coacervates were observed exclusively when at least one of the polypeptides present was racemic (Fig. 1). Although the majority of the work done here utilized a 50/50 random copolymer of *D* and *L* monomers, we have also observed the formation of liquid coacervates when a sequence-controlled polymer of alternating *D* and *L* monomers was used (Supplementary Fig. 3). In contrast, complexes composed only of homochiral polymers (that is, p*L*K*+*p*L*E, p*L*K*+*p*D*E, p*D*K*+*p*L*E, p*D*K*+*p*D*E) formed solid precipitates, regardless of salt concentration.

**Figure 2:**
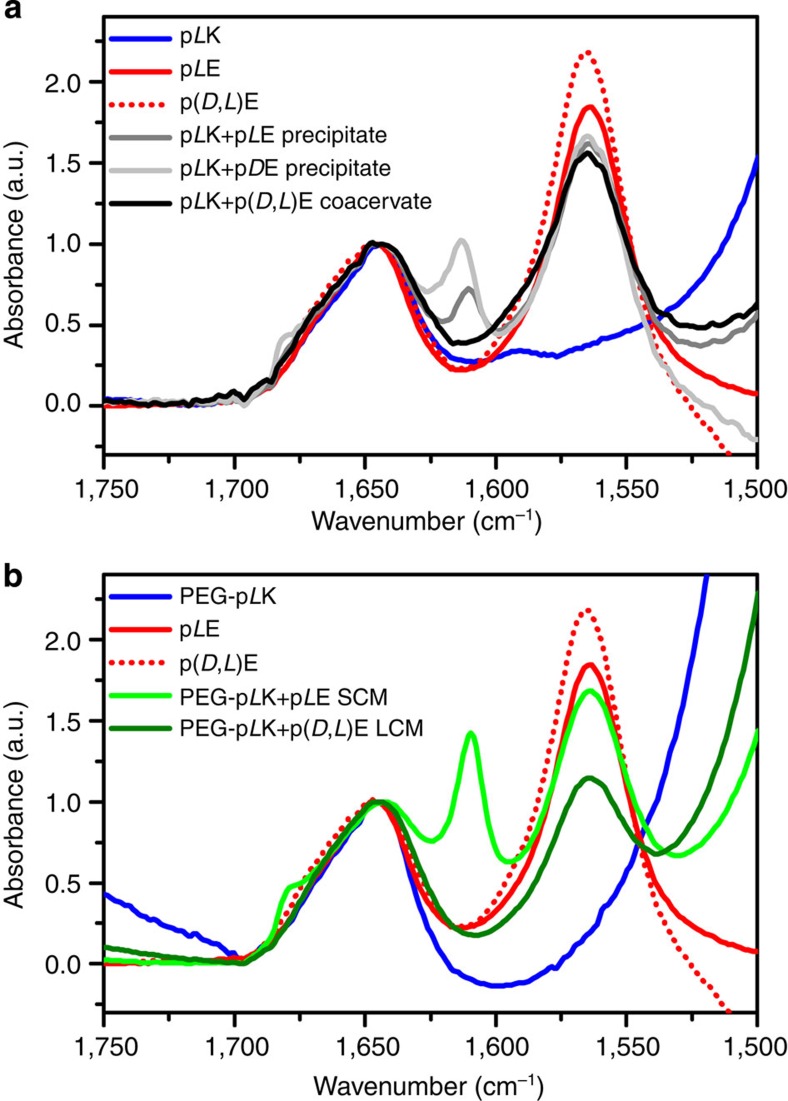
FTIR of polyelectrolyte complexes. FTIR spectra showing the amide I region for (**a**) individual polypeptides and the resulting liquid coacervates and solid precipitates, as well as (**b**) the polypeptides and block copolymers involved in the formation of related LCM and SCM. All samples were prepared in D_2_O. Polypeptides were analysed at a concentration of 10 mM with respect to monomer, liquid coacervates and solid precipitates at a concentration of 6 mM with respect to monomer and 100 mM NaCl, while micellar complexes were prepared at a concentration of 0.186 mM polymer, with no salt. All materials show a peak at 1,644 cm^−1^, characteristic of random coil polypeptide structure. However, the additional peaks associated with aggregated β-strands are present for the solid precipitates and SCMs. For solid precipitates formed from polypeptides with matching chirality (p*L*K*+*p*L*E, p*D*K*+*p*D*E), the main peak is located at 1,611 cm^−1^ and is shifted to 1,613 cm^−1^ for opposite chirality (p*L*K*+*p*D*E, p*D*K*+*p*L*E). For SCMs, this peak is located at 1,610 cm^−1^. An additional low-intensity peak is also present near 1,680 cm^−1^. The signal for the carbonyl stretching of the glutamic acid can also be observed at 1,564 cm^−1^. Micelles were prepared using a polyethylene glycol-p*L*K block copolymer with an average *N*=50 and either p*L*E with *N*=50 for SCMs or *N*=100 for LCMs.

To understand the secondary structure of the polypeptides within the resultant solid or fluid complexes, we employed transmission Fourier transform infrared spectroscopy (FTIR, Bruker Vertex 70 spectrometer). The location of the amide I carbonyl stretching vibration provides a characterization of polypeptide secondary structure^33^,^34^,^37^. As can be seen in Fig. 2a, all samples (that is, individual polypeptides, liquid coacervates and solid precipitates) display a peak at 1,644 cm^−1^, characteristic of a random coil chain configuration (complete FTIR data and discussion are available in Supplementary Fig. 4 and Supplementary Note 1, respectively)^33^,^34^,^37^. This random coil structure was expected for the individual, charged polypeptides, and is consistent with previous characterization of the polymer structure in liquid coacervates closely approximating that of an ideal Gaussian chain^26^ For the solid precipitates, we observe additional peaks that are characteristic of β-strands and amyloids^33^,^34^,^37^. This signal was present at 1,611 cm^−1^ for solid precipitates formed from polypeptides with matching chirality (p*L*K*+*p*L*E, p*D*K*+*p*D*E) and at 1,613 cm^−1^ for complexes formed from polypeptides with opposite chirality (p*L*K*+*p*D*E, p*D*K*+*p*L*E). An additional, low-intensity peak near 1,680 cm^−1^ is also attributable to the presence of β-sheet structure. The blue-shift observed in the main amide I band has been attributed to a decrease in the number of peptide-water contacts, implying that complexes with opposite chirality exclude more water^34^,^37^,^38^.

These results demonstrate the correlation between polypeptide chirality, polymer conformation and the fluid or solid nature of the resulting complex. While the random coil structure of an individual polypeptide homopolymer is a consequence of electrostatic repulsion from the charged side chains, neutralization of these charges allows attractive hydrogen-bonding interactions to dominate, resulting in the formation of close-packed polypeptide secondary structure (that is, α-helix or β-sheet). For the case of homochiral polypeptides, the electrostatic interaction of oppositely charged side chains facilitates alignment of the peptide backbone and the formation of hydrogen-bonded β-strand structures where backbone hydrogen bonds replace surface-bound water that is released during the aggregation of the polymer^33^. However, the presence of a racemic polypeptide prevents the formation of compact protein-like secondary structures and appears to limit both the extent of complexation and the expulsion of water from the polymer rich phase, while maintaining a Gaussian coil conformation^26^.

### Stability of polyelectrolyte complexes against salt and urea

**Figure 3:**
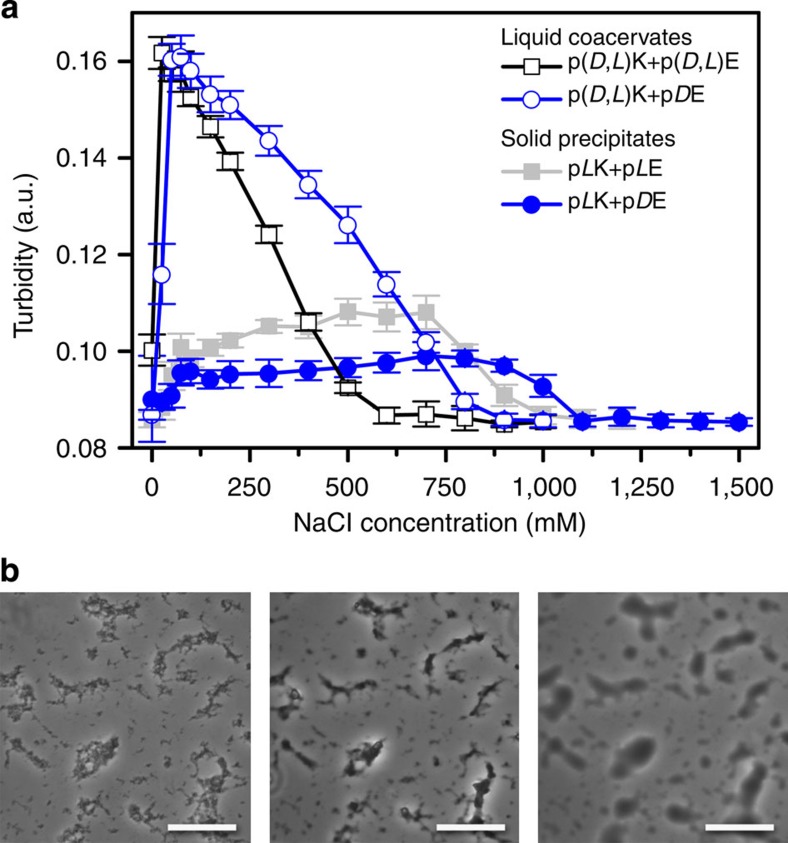
Salt and urea stability of polyelectrolyte complexes. (**a**) Turbidity as a function of NaCl concentration for various liquid coacervates (open symbols) and solid precipitates (solid symbols) prepared at 1 mM total residue concentration and pH=7.0. Error bars are the s.d. from triplicate measurements. (**b**) Optical micrographs showing the transition from solid precipitate to liquid coacervate for p*L*K+p*L*E complexes with increasing urea concentration. Scale bars, 25 μm.

We also investigated the stability of the various solid and fluid polyelectrolyte complexes with respect to salt. Turbidimetric measurements for liquid coacervates showed a characteristic increase in the amount of complex formation at low salt concentrations, followed by a decrease in complex formation up to a critical salt concentration, above which no phase separation is observed (Fig. 3a, Supplementary Fig. 5a and Supplementary Note 2). Taking into account the dependence of polymer chain length on the critical salt concentration (see Supplementary Table 2 and Supplementary Note 2)^18^,^19^, we observed a significant decrease in stability against salt (lower stability means that complexes dissociate and dissolve at lower salt concentrations) when fully racemic complexes were formed, as opposed to complexes with only a single racemic polymer (for example, p(*D*,*L*)K+p(*D*,*L*)E versus p(*D*,*L*)K+p(*D*)E). However, coacervates were significantly less stable in salt as compared with solid precipitates, and solid complexes formed from polypeptides with opposite chirality showed even higher salt stability than those of matched chirality (for example, p*L*K*+*p*L*E, p*L*K*+*p*D*E) (Fig. 3a, Supplementary Fig. 5b and Supplementary Note 2). These variations as a function of polypeptide chirality suggest that electrostatic interactions are not solely responsible for the stability of the complex, but also van der Waals stereoregular interactions and hydrogen bonding.

In polypeptides, short-range hydrogen bonding plays a critical role in defining stable structural motifs. Therefore, we also investigated the stability of our complexes in urea, a denaturant that interacts preferentially with the peptide backbone and disrupts hydrogen bonds^39^. While urea did not affect the formation or stability of liquid coacervates, even at very high concentrations, a much more dramatic effect was observed for solid precipitates. The addition of sufficient quantities of urea resulted in the melting of solid precipitates into a coacervate-like liquid (Fig. 3b, Supplementary Fig. 6 and Supplementary Note 2), a transition that had only been previously observed in relation to charge-driven phenomena in polyelectrolyte complexes^24^. Furthermore, the trends in stability observed as a function of urea matched those observed for salt (Supplementary Table 2), implying that the variations in salt stability are attributable to the strength of hydrogen-bonding effects. This conclusion is supported by the observed differences in polypeptide secondary structure, where hydrogen-bonded β-sheets provide increased stability against salt dissolution for solid complexes as compared with liquid coacervates. Furthermore, the differences in stability observed for solid complexes of differing chirality are supported by the FTIR data (Fig. 2a and Supplementary Fig. 4d), suggesting that complexes with opposite chirality incorporate less water^34^,^37^,^38^, and thus denaturant, from their surfaces, enhancing the stability of hydrogen-bonding interactions.

### Chirality effects in micellar polyelectrolyte complexes

**Figure 4:**
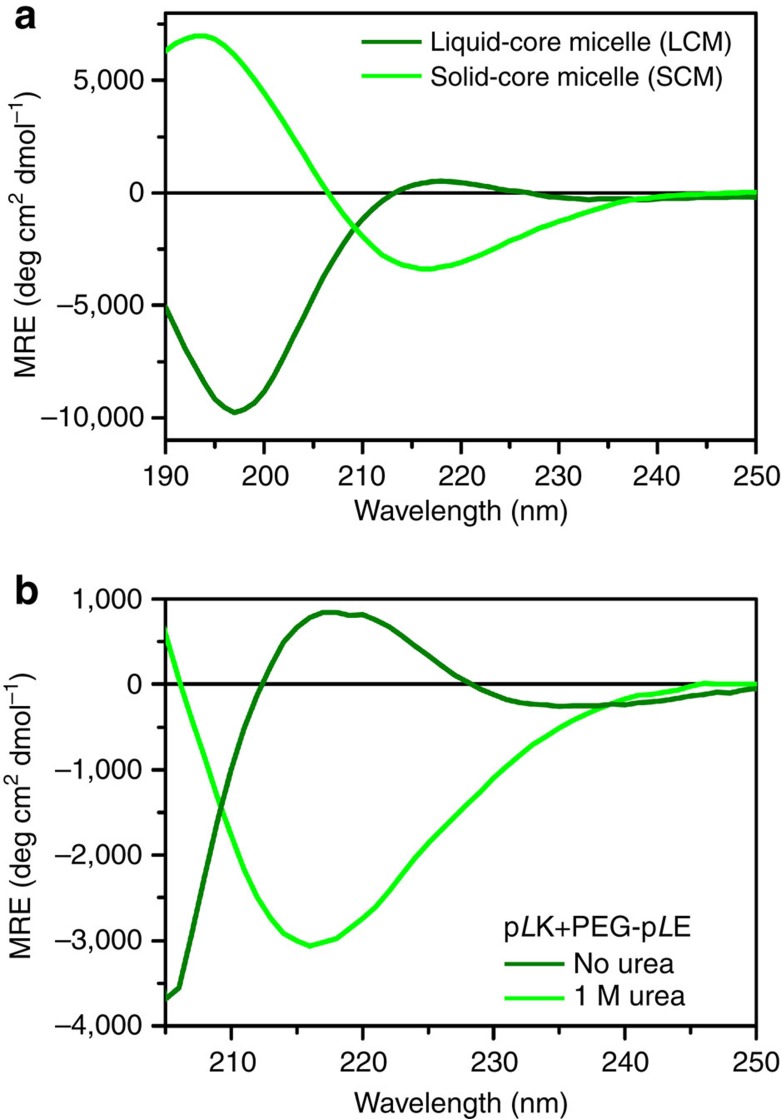
Secondary structure of micellar complexes. CD spectra for (**a**) LCM (dark green) and SCM (light green) micelles formed from PEG-p*L*K+p(*D*,*L*)E and PEG-p*L*K+p*L*E, respectively, and (**b**) SCMs in the absence (light green) and presence of 1 M urea (dark green), respectively. LCMs show a random coil structure, while SCMs display β-sheet character in the absence of urea, but convert to a random coil structure in the presence of 1 M urea, suggestive of a LCM. LCMs were prepared at a polymer concentration of 0.01 mM total polymer concentration, while SCMs were prepared at 0.0125, mM with an average *N*=100 for the micelles in **a**, and both LCMs and SCMs were prepared at a total polymer concentration of 0.04 mM with an average *N*=50 for **b**.

On the basis of experience with bulk complexes and previous reports on polyelectrolyte complex micelles formed using racemic polypeptides^12^, we extended our investigation to include micellar complexes formed from the complexation of a homopolymer polypeptide with a diblock copolymer with one peptide block (Supplementary Fig. 2). In agreement with our results from bulk experiments, micelles formed from electrostatic complexation involving at least one racemic polypeptide (for example, PEG-p*L*K with p(*D*,*L*)E) resulted in the formation of coacervate- or liquid-core micelles (LCMs), as described in the literature^6^,^8^,^12^. These LCMs are highly monodisperse (*R*_h_=27.1 nm, polydispersity=0.074), as characterized by dynamic light scattering (DLS, Brookhaven Instruments BI-200SM, see Supplementary Table 3) and also similar in size to polypeptide-based polyelectrolyte complex micelles of comparable polymer chain lengths observed previously^40^. Secondary structure analysis via both FTIR (Fig. 2b) and circular dichroism spectroscopy (CD, Fig. 4a) shows a random coil conformation, in agreement with the results obtained for bulk complexes. However, the structures formed from the complexation of homochiral polypeptides (for example, PEG-p*L*K with p*L*E) resulted in micelles that were larger, more polydisperse (*R*_h_=32.8 nm, polydispersity=0.117), and displayed β-sheet character (Figs 2b and 4a). We designate these complexes as solid-core micelles (SCMs) based on the behaviour of similar complexes in bulk. Interestingly, while the FTIR spectra for both LCMs and SCMs display the same features as the analogous bulk materials, the location of the β-strand peak for SCMs is slightly red-shifted compared with the bulk, from 1,611 to 1,610 cm^−1^. This shift suggests that the confinement imposed by the micellar structure could enhance packing of the β-sheet network, compared with the bulk, because of forced chain alignment at the polyelectrolyte core-PEG corona interface^37^.

CD enabled characterization of the urea-induced polypeptide structural changes in the micellar systems (Fig. 4b). While our initial SCM sample displayed 100% β-sheet character, as calculated using CD basis spectra and evidenced by the characteristic minimum at 215 nm (Supplementary Fig. 7a), the addition of 1 M urea caused an unfolding transition, suggested by the appearance of a maximum near 220 nm and a minimum below 205 nm. Spectral fitting suggested a micelle with 99.8% random coil structure (Supplementary Fig. 7c,d, Supplementary Note 3). As in the case of our bulk complexes, the observed transition to a random coil structure is reminiscent of liquid complexes or LCMs (Fig. 4a and Supplementary Fig. 7b), suggesting that hydrogen bonding is a key determinant of the solid or liquid character of polypeptide-based polyelectrolyte complexes. Furthermore, this provides a way of independently manipulating the solid or liquid character of complexes in bulk or in domains. The moduli and self-healing character of ordered phases based on polyelectrolyte complexation will depend on the phase selection of the domains^10^.

**Figure 5:**
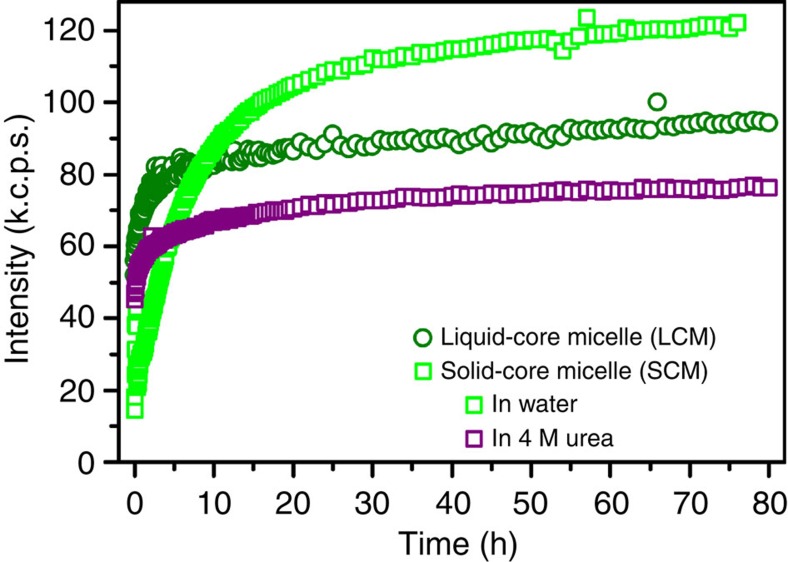
Kinetics of micelle formation. Total scattered intensity as a function of time for LCMs (circles) and SCMs (squares) depicting the timescale for equilibration. LCMs in water equilibrate relatively quickly, reaching 72% of their equilibrium value within 1 h, while SCMs in water show much slower kinetics, on the order of days; only reaching 25% of their equilibrium value in 1 h. However, the addition of 4 M urea to a SCM sample to disrupt hydrogen bonding between the polypeptides enables fast equilibration, similar to that of LCMs (74% of equilibrium value in 1 h). Micelles were prepared at a polymer concentration of 0.01 mM total polymer concentration using a polyethylene glycol-p*L*E block copolymer with an average *N*=50 and either p*L*K with *N*=100 for SCMs or p(*D*,*L*)K with *N*=100 for LCMs.

Commensurate with phase selection and materials properties, solid and liquid complexes also display differences in the kinetics of assembly. Light scattering measurements were utilized to track the formation and equilibration of both LCMs and SCMs as a function of time (Fig. 5). The dynamic nature of the liquid coacervate core enabled the fast equilibration of LCMs. After 1 h, LCMs had reached 72% of their equilibrium size. This is in contrast to SCMs, where the hydrogen-bonded, β-sheet solid core is more resistant to chain rearrangement and was only able to attain 25% of its equilibrium size after 1 h. We confirmed the dominant effect of hydrogen bonding in preventing chain rearrangement by further examining a presumed SCM sample that was formed in the presence of 4 M urea. Here disruption of the hydrogen bonds by urea enabled recovery of the liquid-like timescales for chain rearrangement, attaining 74% of the equilibrium value after 1 h. These data provide further support for the hypothesis that the physical state of the system (that is, SCM or LCMs) is determined by the presence of hydrogen bonds, which favour a more solid-like structure by creating a higher barrier for chain rearrangement and thus slower equilibration kinetics.

### Molecular dynamics investigation of chirality effects

**Figure 6:**
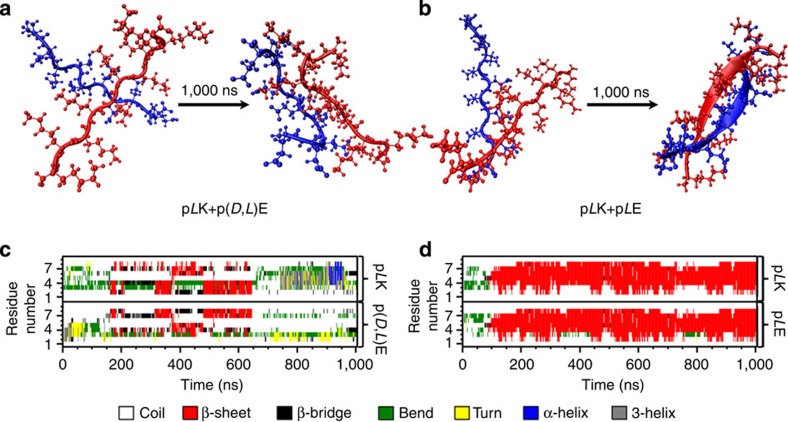
MD simulations of polyelectrolyte complexes. Visualization and residue maps indicating polypeptide secondary structure from representative MD simulations for two pairs of poly(lysine) and poly(glutamic acid) peptides, *N*=10. Polypeptides are initially equilibrated in a random coil conformation and then allowed to complex for 1,000 ns. (**a**) A representative simulation of homochiral p*L*K complexing with racemic p(*D*,*L*)E indicates preservation of a mostly random coil structure, as would be expected for liquid coacervates, while (**b**) homochiral polypeptides p*L*K with p*L*E shows the evolution of β-strand structure expected for a solid precipitate. Map of secondary structure as a function of time for (**c**) p*L*K+p(*D*,*L*)E and (**d**) p*L*K+p*L*E.

**Figure 7:**
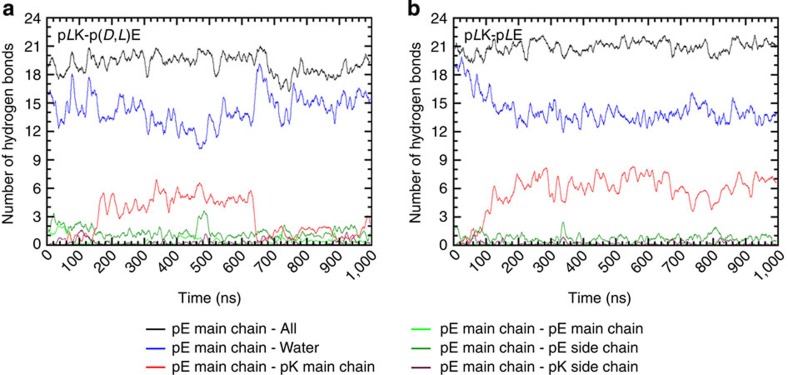
Hydrogen bonding in polyelectrolyte complexes. Quantification of the number of hydrogen bonds formed as a function of time during a 1,000 ns MD simulation of complex formation between (**a**) p*L*K+p(*D*,*L*)E and (**b**) p*L*K+p*L*E.

To further investigate the different intermolecular interactions that give rise to the various solid and liquid complexes, we performed molecular dynamics (MD) calculations. Multiple independent simulations were run for a 10-residue p*L*K interacting with a 10-residue p(*D*,*L*)E, while one simulation was run for a 10-residue p*L*K interacting with a 10-residue p*L*E. All simulations were performed in 173 mM NaCl over the course of 1,000 ns. In all systems, the peptides formed an electrostatic complex in less than 10 ns, adopting a disordered structure. Around 150 ns, the p*L*K+p*L*E system began to form a parallel β-sheet in the centre of the peptides, which was fully formed by 200 ns (Fig. 6). This structure remained stable throughout the duration of the simulation, with the exception of small fluctuations at the termini. The formation of a stable β-sheet structure allowed the peptides to bind more tightly, reducing the centre of mass distance to about 0.2 nm. However, the p*L*K+p(*D*,*L*)E system shown in Fig. 6 formed a less compact structure with a centre of mass distance varying between 0.3 and 0.9 nm. These peptides remained in mostly coil, bend and turn conformations, with only rapidly transient β-sheet formation observed for durations of less than 100 ns, and mostly in a region of the p(*D*,*L*)E peptide where a sequence of three consecutive *L* amino acids was present (Fig. 6). Furthermore, the analysis of backbone hydrogen-bonding interactions indicated that the unstructured p*L*K+p(*D*,*L*)E system, corresponding to the liquid coacervate complexes, showed a preferential interaction with water and formed very few interpeptide hydrogen bonds (Fig. 7a). In contrast, the dense β-sheet p*L*K+p*L*E complex satisfied nearly all of its backbone hydrogen bonds through peptide-peptide interaction (Fig. 7b). Averaging multiple independent enactments of these 1,000-ns simulations with some variability of initial conditions, the p*L*K+p*L*E system had an average β-sheet content over all residues of 61.3%, compared with 11.4% for the average of the p*L*K+p(*D*,*L*)E simulations. These results provide direct support of our experimental observations, confirming the structural modes of interaction between the various oppositely charged polypeptides and demonstrating the key role that chirality and hydrogen bonding play in determining the structural and physical state of polypeptide-based polyelectrolyte complexes.

## Discussion

In summary, the polypeptide chirality not only determines the physical state of the resulting polyelectrolyte complexes (that is, liquid or solid), but also defines the strength of intermolecular interactions, and thus the material properties. While electrostatic interactions act over long distances, the shorter-range nature of polar hydrogen-bonding forces, combined with steric packing and hydration, provide additional methods for controlling self-assembly. The effects we have presented here raise obvious follow-on questions about the effects of sequence distribution within globally achiral polymers or designed effects that can be created by tailoring sequences of chiral peptides, which we are now pursuing. While sequence specificity in biology controls the three-dimensional assembly of proteins, we propose that patterns of chirality could have significant implications for tailoring of material properties without otherwise altering the chemical composition of polypeptide-based materials. For instance, this type of control could be utilized to tailor the rheological response of bulk materials and formulate delivery systems with controlled water content. Furthermore, coupling this type of polar and electrostatically guided self-assembly with more complex molecular architectures, as in the block copolymer systems described here, enables the creation of interesting classes of new materials with novel self-assembling structures, functionality and responsiveness^41^. We note, too, that while this work clarifies the role of chirality on phase selection in complexes of charged polypeptides, other polyelectrolyte complex systems also form both solid and fluid phases^24^. We suggest that the causes of solid phase formation in polyelectrolyte complexes are to be found in short-range forces, which may be influenced by tacticity, hydration packing and other factors, acting in concert with longer range electrostatic forces.

## Methods

### Materials

Polypeptides were obtained either from Alamanda Polymers Inc. and used directly, without further purification, or were synthesized in house using *N*-carboxyanhydride polymerization^42^. In a previously published article^19^, we reported results on a polypeptide that we said was p*L*E; after communication with the supplier (Alamanda Polymers Inc.) and performing further characterization, it came to our attention that the polymers in this previous work were not optically pure *L* but contained a number of *D* repeating units and should therefore have been referred to as p(*D*,*L*)E. A correction of this error has now been published^43^ Identification of this issue led to the current work in which we have retested a wide range of conditions and have been unable to formulate liquid coacervates using homochiral polypeptides in any instance.

The degree of polymerization for the prepared polymers was obtained via ^1^H NMR. Gel permeation chromatography (Waters) coupled with refractive index (Optilab UT-rex, Wyatt Technologies) and light scattering detectors (miniDAWN Treos, Wyatt Technologies) was used to characterize the polydispersity of the samples (Supplementary Table 1). CD (Jasco J-815 CD Spectrometer) was used to confirm both the random coil structure and the homochiral or racemic composition of the individual polypeptides (Supplementary Figs 1 and 2).

Here we abbreviate poly(glutamic acid) as pE in general, or the different chiral polymers as p*L*E, p*D*E, p(*D*,*L*)E in specific, taking advantage of the single-letter abbreviation strategy for amino acids. Similarly, we refer to poly(lysine) as pK in general, or p*L*K, p*D*K, p(*D*,*L*)K in specific. This naming convention also allows referring to *D*-amino acids using a lower-case letter (that is, pk rather than p*D*K). This strategy will be useful for the investigation of sequence-controlled polymers of *D* and *L* amino acids (for example, (Kk)_14_W as in Supplementary Fig. 3), but for the current discussion of homochiral or random copolymers we will explicitly specify the *D* or *L* chirality of the polymer for clarity.

pE is a weak acid with a pKa around pH 4.3, while pK is a weak base with a pKa around pH 10.0 (refs 44, 45). We make the assumption that, at solution conditions at least two pH units away from the pKa, the polypeptides are fully charged. Stock solutions were prepared gravimetrically using MilliQ water (resistivity of 18.2 MΩ-cm, Millipore) at a concentration of 10 mM with respect to the number of monomers (that is, the number of acid or base groups) present in solution and then adjusted to pH 7.0 using concentrated solutions of HCl and NaOH, as needed. Monomer concentration was chosen as the experimental basis to easily enable direct stoichiometric comparison of the number of positively and negatively charged units present in solution. Stock solutions of 2 M sodium chloride (NaCl, ACS reagent, ≥99%, Acros Organics) and 8 M urea (Bioreagent, Sigma) were prepared gravimetrically and adjusted to pH 7.0, as above.

### Preparation of bulk polyelectrolyte complexes

Complexation was performed using stoichiometric quantities of positive and negatively charged polypeptides at a total residue concentration of 1 mM at pH 7.0, in the presence of varying concentrations of NaCl and urea. Under these conditions, it is a reasonable approximation to describe all of the residues on both polypeptides as charged. All polymers used for the preparation of bulk complexes have an approximate degree of polymerization *N*=100. Samples were prepared by first mixing a concentrated solution of NaCl with MilliQ water in a microcentrifuge tube (1.5 ml, Eppendorf). Other additives such as concentrated urea were also added at this stage, unless otherwise specified. The polyanion (pE) was then added to this mixture, followed by the polycation (pK) to a final volume of 500 μl. The mixture was vortexed for 5 s immediately after the addition of each component to ensure fast mixing. For all experiments, samples were prepared to a final concentration of 1 mM monomers (total cation and anion). Unless otherwise indicated, all bulk complexes (that is, liquid coacervates and solid precipitates) were prepared in 100 mM NaCl. The effect of salt was examined over the range of 0 to 1.5 M NaCl. The effect of urea was examined from 0 to 6.8 M. All samples were prepared immediately before analysis and studied at room temperature (25 °C).

### Preparation of micellar polyelectrolyte complexes

Micelles were formed by mixing either PEG-p*L*K with pE or PEG-p*L*E with pK at an equal charge molar ratio, in water, using the order of operations described above for the homopolymers. Micellar solutions for the DLS experiments were made using PEG-p*L*K and pE, in which the average charged polypeptide segments of each molecule was *N*=100. The micelle formed using PEG-p*L*K and p(*D*,*L*)E was made at a total polymer concentration of 0.07 mM, which was subsequently diluted to 0.01 mM when measured using CD. The micelle formed using PEG-p*L*K and p*L*E was made at a total polymer concentration of 0.05 mM, which was diluted to 0.0125, mM when measured using CD. The micellar urea experiments were performed by complexing PEG-p*L*E with pK at a total polymer concentration of 0.04 mM, with all the charged segments containing an average *N*=50. Micellar solutions for the FTIR experiments were made at a total polymer concentration of 0.19 mM in D_2_O using a PEG-p*L*K block copolymer with an average *N*=50 and either p*L*E with *N*=50 for SCMs or *N*=100 for LCMs.

### Visual characterization of complexes

The resulting complexes formed from homopolymers were imaged within 1 h of preparation. Bright-field and phase contrast optical microscopy (Leica DMI 6000B using Leica AFC image acquisition software) were used to identify the formation of liquid coacervates or solid precipitates, to identify the critical salt concentration, above which no phase separation occurs, and the minimum urea concentration necessary to trigger the transformation between solid precipitates and liquid coacervates. Liquid coacervates appear as small spherical droplets in solution, while precipitates form amorphous solid clusters (Figs 1 and 3 and Supplementary Figs 3 and 6). Imaging was performed using both ultra-low attachment 96-well plates (Costar, Corning Inc.) and glass slides (Fisherbrand).

### Turbidimetry

Turbidity was used to qualitatively measure the extent of complex formation as a function of charge stoichiometry and salt concentration. Turbidity measurements were made using a plate reader equipped with a ultraviolet spectrophotometer (Tecan, Infinite M200 P80). Turbidity was measured at a wavelength of 550 nm and a temperature of 25 °C. None of the polymers absorb light at this wavelength. Turbidity is defined as −ln(*I*/*I*_o_), with *I*_o_=incident light intensity and *I*=intensity of light passed through the sample volume, and is measured in absorption units (a.u.). After preparation, 100 μl of sample was pipetted in triplicate into a 96-well plate (black/clear with lid, BD Falcon) for analysis. Triplicate measurements were made for each well, and all experiments were repeated three times. Error bars on turbidity plots represent the calculated s.d. of the data.

### Characterization of polypeptide secondary structure

Analysis of the secondary structure of the various individual polypeptides, liquid coacervates, solid precipitates and micellar complexes was performed using transmission FTIR spectroscopy. The instrument used was a Bruker Vertex 70 FTIR spectrometer with a DTGS detector. The sample was prepared in D_2_O and held between two 1-mm thick CaF_2_ windows with a 50-μm PTFE spacer. The sample chamber was purged with N_2_ for 10 min before data collection. One hundred and twenty-eight scans were collected from 4,000 to 800 cm^−1^ at a spectral resolution of 2 cm^−1^. Spectra were obtained using a D_2_O sample as a background and were dynamically corrected for atmospheric water by subtracting the signal obtained for the open chamber (exposed to air), taken with a N_2_ background and scaled by a factor of ~−0.018. Data were then normalized such that the minimum baseline signal above the amide I signal (in the range of 1,755–1,700 cm^−1^) was set to zero, and the height of the random coil peak near 1,644 cm^−1^ was set to 1. The location of individual peaks was determined using non-linear least-squares fitting of Gaussian peaks to the features of the spectrum, with baseline correction where necessary.

CD (Jasco J-815 CD Spectrometer) was used to confirm the random coil structure, the chiral nature of the individual polypeptide solutions (Supplementary Figs 1 and 2) and the secondary structure of micellar solutions (Supplementary Fig. 7). All the CD data presented are an average of five scans collected between 250 and 190 nm at room temperature, in a 0.1-cm cuvette. In experiments involving urea, the baseline measurements of the solvent were subtracted from the micellar spectra. The fitting of the CD data was done using a linear combination of poly(lysine) basis spectra^46^ resulting in specific percentages of α-helix, β-sheet and random coil secondary structure.

### Dynamic light scattering

DLS was measured at 90° using a BI-200SM goniometer containing a red laser diode with a wavelength of 637 nm and a TurboCorr digital correlator (Brookhaven Instruments, Holtsville, NY). Brookhaven Instruments DLS software was used to analyse the intensity autocorrelation function using the cumulant method^47^ to obtain characteristic decay rates from which apparent diffusion coefficients were calculated. The Stokes–Einstein equation was used to convert diffusion coefficients to hydrodynamic radii. Polydispersity was obtained from the second order cumulant term.

### MD simulations

MD simulations were performed for 10-residue peptides of poly(glutamic acid) (pE) and poly(*L*-lysine) (p*L*K). Two versions of pE were used: a homochiral *L* version (p*L*E) and a racemic version (p(*D*,*L*)E) with the sequence LLDDDLLLDD, which was chosen by shuffling a sequence with five *L* and five *D* chiral centres. The side chains were fully charged with the N terminus and C terminus consisting of a charged NH_3_^+^ and COO^−^ group, respectively. These were initialized into a β-sheet conformation. One pE chain and one p*L*K chain were placed perpendicular to each other ~1 nm apart in a 6-nm dodecahedral box. The system was then solvated with 4889 TIP3P waters and 16 Na^+^ and Cl^−^ ions (that is, 104 mM NaCl), modelled with the ion parameters from Joung *et al.*^48^ Molecular simulations were run using the GROMACS 4.6.3 simulation package^49^. The energy of the system was minimized for 500 steps without constraints and then for an additional 10,000 steps with all bonds constrained using the steepest descent algorithm. A MD simulation was then run using a step size of 2 fs and the CHARMM22* force field^50^,^51^ for 100 ps at constant volume and a temperature of 298 K. This was continued for 1,000 ns at a constant pressure of 1 bar using the Nose–Hoover^52^ and Parrinello–Rahman^53^ coupling schemes to maintain temperature and pressure, respectively. Electrostatic interactions were performed using a particle mesh Ewald method^54^ with a cutoff of 0.9 nm and a Fourier spacing of 0.33 nm. Hydrogen bonds were constrained using the LINCS^55^ algorithm. The secondary structure was determined using the DSSP (Define Secondary Structure of Proteins) criteria^56^ over an average of four replicate simulations for p(D,L)E with pLK and one simulation for pLE with pLK.

## Author contributions

S.L.P. and L.L. contributed equally to this work. S.L.P. designed and performed the experiments involving bulk polyelectrolyte complexes. L.L. designed and performed the experiments involving micellar polyelectrolyte complexes. S.L.P., L.L., K.Q.H. and M.T. wrote the article. All authors discussed and contributed to the interpretation of the data. M.J.K., C.F.P., L.L. and R.A.K. synthesized the racemic polycations. D.P. and K.A.B. performed proof of concept work. R.A.K. and K.O.M. performed characterization experiments on bulk polyelectrolyte complexes using optical microscopy and turbidity measurements. D.W. performed characterization experiments using CD and DLS on the micellar polyelectrolyte complexes. K.Q.H. designed and performed MD simulations. S.L.P., L.L, D.P., K.Q.H., J.Q., J.K.W., J.J.d.P. and M.T. edited the manuscript. J.J.d.P. supervised the computational work. M.T. supervised the experimental work.

## Additional information

**How to cite this article:** Perry, S. L. *et al.* Chirality-selected phase behaviour in ionic polypeptide complexes. *Nat. Commun.* 6:6052 doi: 10.1038/ncomms7052 (2015).

## Supplementary Material

Supplementary Information Supplementary Figures 1-7, Supplementary Tables 1-3, Supplementary Notes 1-3, and Supplementary References
