# Supplementary material for: Chirality-selected phase behaviour in ionic polypeptide complexes
Source: Nat Commun. 2015 Jan 14;6:6052. doi: 10.1038/ncomms7052 (PMC4309419; doi:10.1038/ncomms7052)
Supplement: Supplementary Information — Supplementary Figures 1-7, Supplementary Tables 1-3, Supplementary Notes 1-3, and Supplementary References [file ncomms7052-s1.pdf]

Supplementary figures

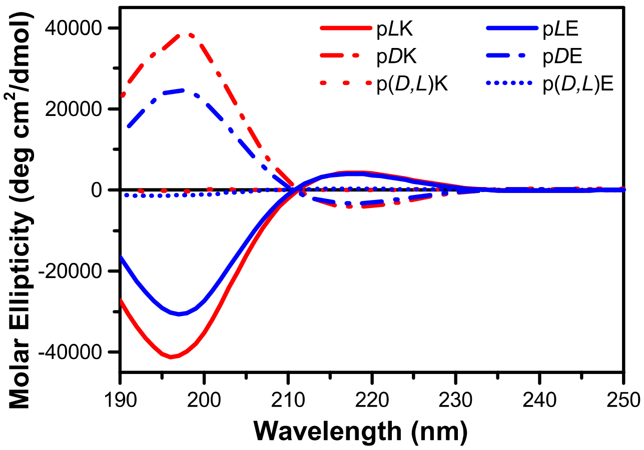

Supplementary Fig. 1 | Secondary structure of homopolymers. CD spectra for homopolymer polypeptides in water (10 mM with respect to individual residues). Poly(lysine) is shown in red, poly(glutamic acid) is shown in blue. A strong negative peak between 195 – 200 nm is indicative of a random coil *L*-polypeptide while a positive peak results for *D*-polypeptides. Racemic polypeptides show very little signal due to cancellation of the signal contributions from the *L* and *D* amino acids.

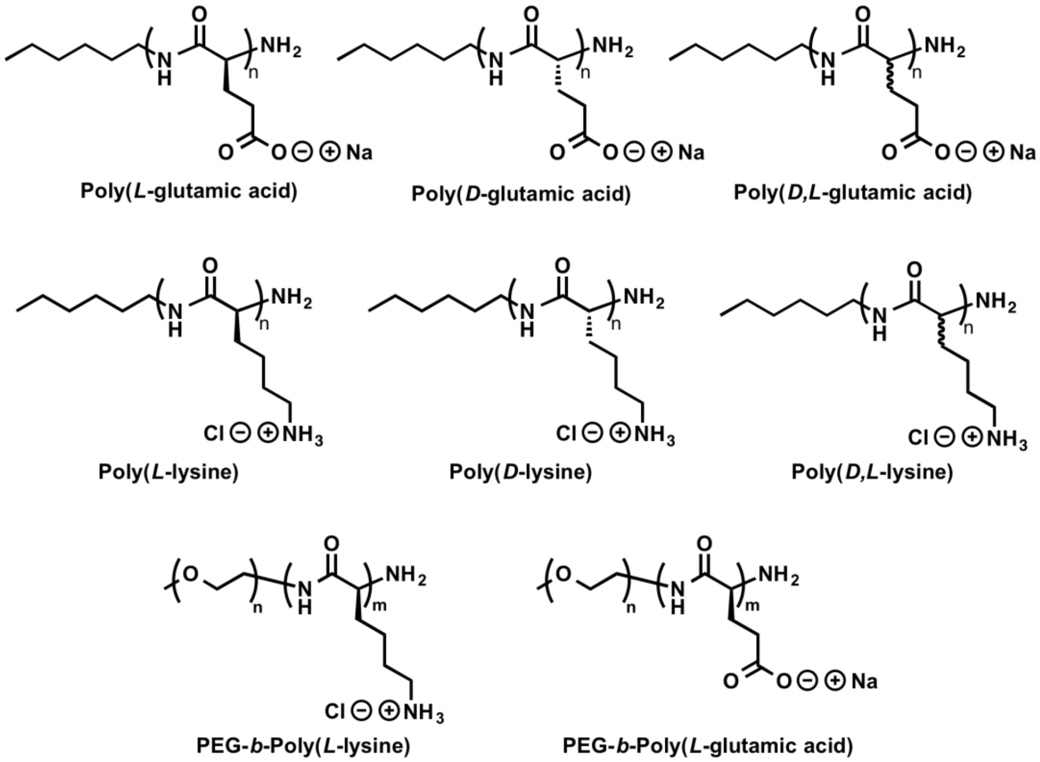

Supplementary Fig. 2 | Chemical structures of polyelectrolytes. Chemical structure of p*L*E, p*D*E, p(*D,L*)E, p*L*K, p*D*K, p(*D,L*)K, as well as PEG-p*L*K and PEG-p*L*E.

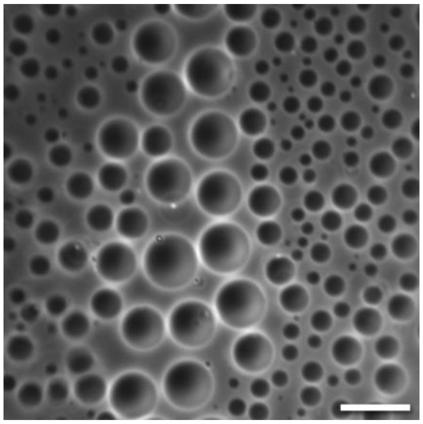

Supplementary Fig. 3 | Microscopy of sequence controlled complexes. Optical micrographs showing the liquid nature of complex coacervates formed from sequence-controlled polymers of alternating *D* and *L* monomers (*i.e.*, $(Kk)_{14}W$ and $EW(Ee)_{14}e$ where K and E indicates an *L* monomer and k and e indicate a *D* monomer, and a tryptophan W was added for concentration determination) at a total monomer concentration of 6.8 mM and 100 mM NaCl. Scale bar is 25 µm.

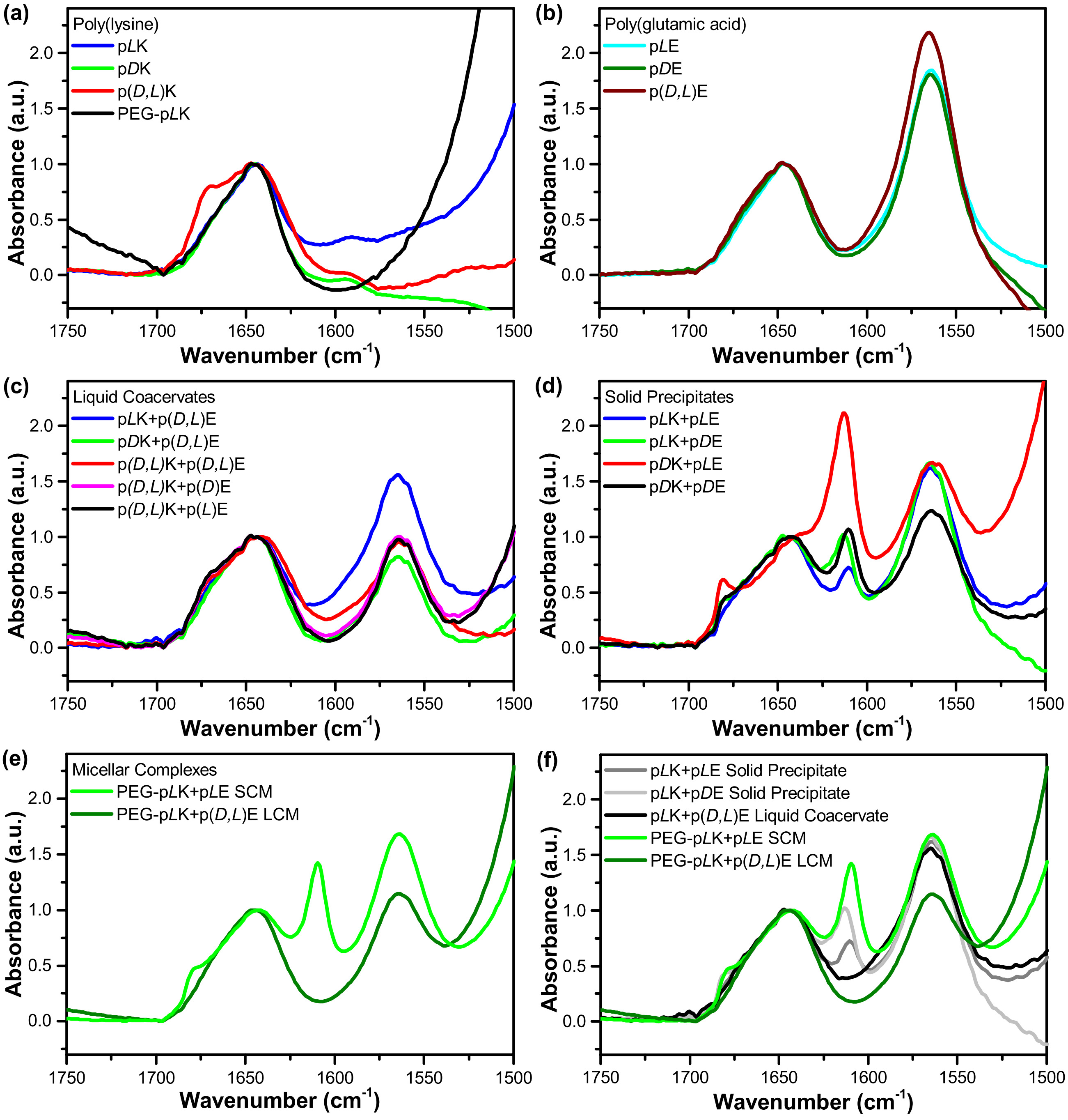

Supplementary Fig. 4 | FTIR of polyelectrolytes, polyelectrolyte complexes and micellar polyelectrolyte complexes. Transmission FTIR spectra showing the amide I region for the individual **(a)** poly(lysine) and **(b)** poly(glutamic acid) polymers, **(c)** liquid coacervates, **(d)** solid precipitates, **(e)** micellar complexes, and **(f)** a comparison of the various solid and liquid, bulk and micellar complexes. All samples were prepared in D_2O, and unless specified, polypeptides with a degree of polymerization N = 100 were used. Polypeptides were analyzed at a concentration of 10 mM with respect to monomer, liquid coacervates and solid precipitates at a concentration of 6 mM with respect to monomer and 100 mM NaCl, while micellar complexes were prepared at a concentration of 0.186 mM polymer, with no salt. Micelles were prepared using PEG-p*L*K with an average N = 50 and either p*L*E with N = 50 for SCMs or N = 100 for LCMs.

(a) Poly(lysine) shows a single peak at 1644 cm^{-1}, characteristic of a random coil structure. **(b)** Glutamic acid shows an identical backbone signal, as well as a second peak at 1564 cm^{-1}, associated with the carbonyl stretching of the side chain. No variations in the FTIR signal were observed as a function of varying chirality. **(c)** Liquid coacervates show a similar random coil structure. **(d)** However, the spectra for solid precipitates contain signals for both random coil polypeptides and aggregated β-strands. For complexes formed from polypeptides with matching chirality (p*L*K+p*L*E, p*D*K+p*D*E) the main peak is located at 1611 cm^{-1}, and is shifted to 1613 cm^{-1} for opposite chirality (p*L*K+p*D*E, p*D*K+p*L*E), suggesting a decrease in the size of the β-sheet network.[1] An additional low intensity peak is also present near 1680 cm^{-1}. **(e)** The spectra for solid- and liquid-core micelles (SCM, LCM) display the same features as the analogous bulk materials, but the β-strand peak location for the SCM is shifted to 1610 cm^{-1}, suggesting a more extended β-sheet network than for the bulk materials.

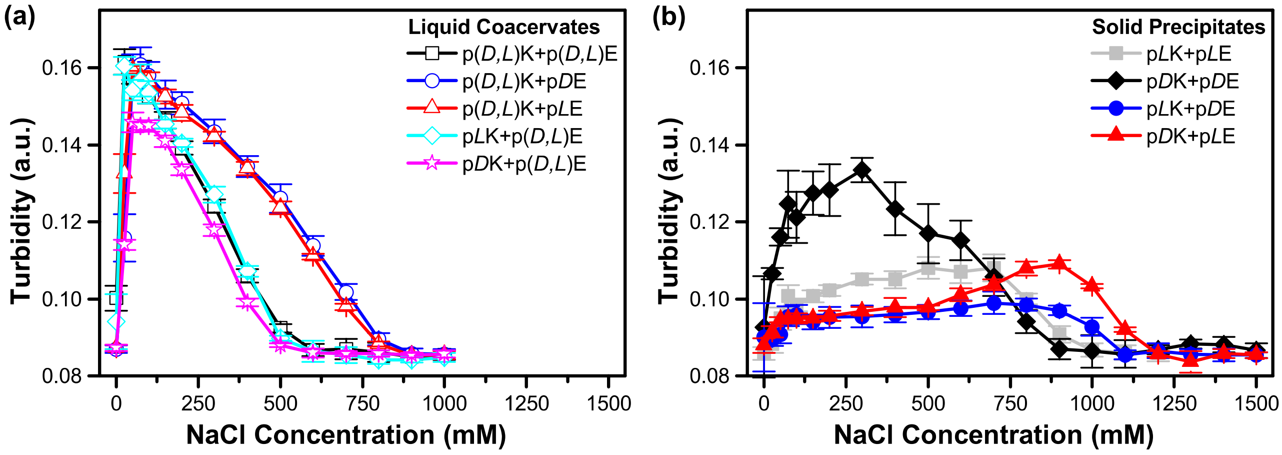

Supplementary Fig. 5 | Salt stability of polyelectrolyte complexes. Turbidity as a function of NaCl concentration for various **(a)** liquid coacervates and **(b)** solid precipitates prepared at 1 mM total residue concentration and pH = 7.0.

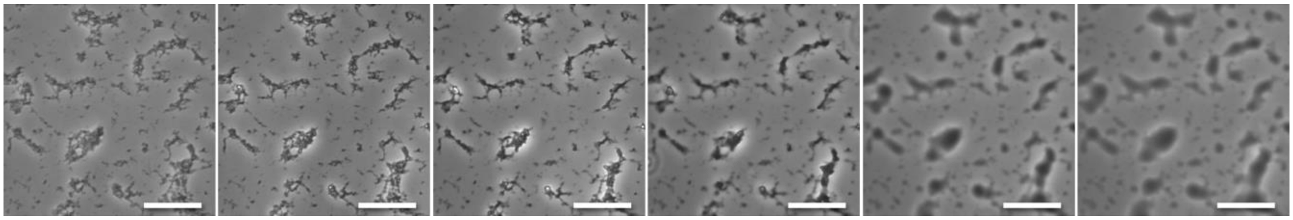

Supplementary Fig. 6 | Solid complexes in the presence of urea. Optical micrographs showing the transition from solid precipitate to liquid coacervate for p*L*K+p*L*E complexes with increasing urea concentration. Scale bars are 25 µm.

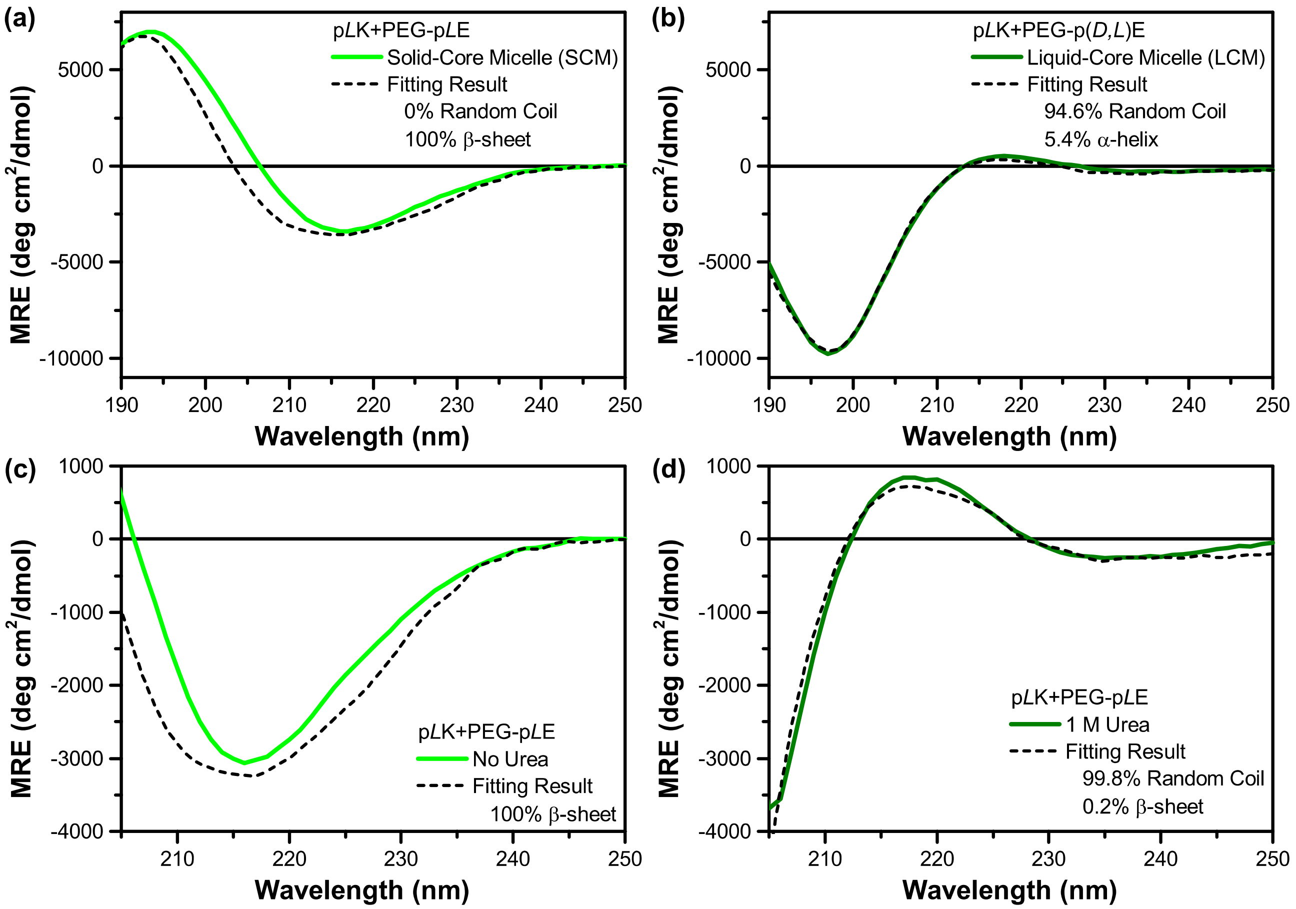

Supplementary Fig. 7 | Analysis of micellar secondary structure. CD spectra and structural fits from basis data for **(a)** solid-core micelles (SCMs, 100% β-sheet), **(b)** liquid-core micelles (LCMs, 94.6% random coil, 5.4% α-helix), and SCMs formed from PEG-p*L*E+p*L*K in the **(c)** absence and **(d)** presence of 1 M urea, respectively. The micelles in **(c)** show a β-sheet character characteristic of SCMs in the absence of urea (100% β-sheet), while the 1 M urea results in **(d)** show the near complete conversion to a random coil structure (0.2% β-sheet, 99.8% random coil), suggestive of a liquid-core micelle (LCM). Micelles were prepared at a polymer concentration of 0.01 mM total polymer concentration for **(a)**, 0.0125 mM for **(b)**, and 0.04 mM for **(c)** and **(d)**. Polypeptides were used with N = 100 for **(a)** and **(b)** and N = 50 for **(c)** and **(d)**.

Supplementary Table 1 | Polymer characterization. Characteristics of the polypeptides used in this study. M_n and degree of polymerization were determined by ^{1}H NMR (Bruker). Polydispersity was determined by GPC (Waters) and static light scattering (Wyatt Technologies).

Sample	Molecular Weight (M_n g/mol)	Polydispersity (M_w/M_n)	Polypeptide Degree of Polymerization (N)
p*L*K50[a]	9,100	1.04	55
p*L*K100*[a]	19,100	1.30	116
p*D*K100[b]	21,800	1.03	104
p(*D,L*)K100*[a]	24,500	1.22	149
p*L*E50[c]	7,700	1.01	51
p*L*E100[c]	14,300	1.01	95
p*D*E100[c]	15,700	1.01	104
p(*D,L*)E100[c]	14,800	1.02	98
PEG-p*L*K50[a]❖	13,400	1.09	51
PEG-p*L*K100[a]❖	21,600	1.07	101
PEG-p*L*E50[c]❖	12,100	1.004	47

* The polymer was synthesized in-house via NCA polymerization.[2] All other polymers were purchased from Alamanda Polymers Inc.

[a] Chloride salt. [b] Bromide salt. [c] Sodium salt.

❖5K PEG.

Supplementary Table 2 | Molecular weight scaled salt and urea stability. Characteristics of the polyelectrolyte complexes used in this study. Average degree of polymerization was calculated from the numbers reported in Supplementary Table 1. The critical salt concentration is the concentration of NaCl at which no phase separation is observed. The critical urea concentration corresponds to the level of urea required to transform a solid precipitate into a liquid coacervate. Raw data represents the direct measurement of the critical concentration. Scaling as a function of polymer molecular weight was also utilized to clarify the effects of variations in molecular weight.

Sample	Average Degree of Polymerization (N)	Critical Salt Concentration (mM)		Critical Urea Concentration (M)	
		Raw Data	MW Scaled	Raw Data	MW Scaled
p*L*K+p*L*E	106	1000	862	4.0	3.4
p*L*K+p*D*E	110	1100	948	6.8	5.9
p*D*K+p*L*E	100	1200	1154	6.8	6.5
p*D*K+p*D*E	104	900	865	2.5	2.4
p*L*K+p(*D,L*)E	107	600	517	--	--
p*D*K+p(*D,L*)E	101	600	577	--	--
p(*D,L*)K+p(*D,L*)E	124	600	403	--	--
p(*D,L*)K+p*D*E	127	900	604	--	--
p(*D,L*)K+p*L*E	122	900	604	--	--

Supplementary Table 3 | Characterization of micellar complexes. Results from the dynamic light scattering analysis of liquid-core (LCM) and solid-core micelles (SCM).

Sample	Type of Micelle	R_h (nm)	Polydispersity
PEG-p*L*K+p(*D,L*)E[a]	LCM	27.1	0.074
PEG-p*L*K+p*L*E[b]	SCM	32.8	0.117

Total polymer concentration of [a] 0.07 mM and [b] 0.05 mM.

Supplementary Notes

Supplementary Note 1: Characterization of polypeptide secondary structure.

The location of the amide I carbonyl stretching vibration provides a characterization of polypeptide secondary structure. Supplementary Fig. 4 shows a comparison of the FTIR spectra for each of the individual polypeptides, solid precipitates, liquid coacervates, and micellar complexes. All samples (*i.e.*, polypeptides, liquid coacervates, and solid precipitates) display a peak at 1644 cm^{-1}, characteristic of a random coil structure.[1,3] Furthermore, no variations in the FTIR signal were observed as a function of chirality for the individual polypeptides (Supplementary Figs. 4a and 4b). In addition to the random coil peak at 1644 cm^{-1} associated with the peptide backbone carbonyl stretch, an additional signal is observed at 1564 cm^{-1} for the side chain stretching of the glutamic acid carbonyl (Supplementary Fig. 4b). The spectra for liquid coacervates matched the random coil structure of the individual polypeptides very closely, again with no variations as a function of chirality. This random coil structure is expected for the individual, charged polypeptides, and is consistent with previous characterization of the polymer structure in liquid coacervates closely approximating that of an ideal Gaussian chain.[4] However, for the solid precipitates, we observe signals characteristic of desolvated β-strands and amyloids.[3,5-7] The main amide I peak is present at 1611 cm^{-1} for solid precipitates formed from polypeptides with matching chirality (p*L*K+p*L*E, p*D*K+p*D*E), and is shifted to 1613 cm^{-1} for complexes formed from polypeptides with opposite chirality (p*L*K+p*D*E, p*D*K+p*L*E). This type of blue-shift in the amide I band has been attributed to decrease in the size of the β-sheet network.[1] An additional, low intensity peak near 1680 cm^{-1} is also attributable to the presence of β-sheet structure. Very similar trends are observed for polypeptides present as block copolymers, and the resulting solid-core and liquid-core micellar complexes (SCM, LCM). Interestingly, the main β-sheet peak for the SCMs is located at 1610 cm^{-1}. This red shift in the peak location, compared to the bulk materials suggests that the confinement effects present within a micellar structure may enhance the formation of an extended β-sheet network.[1]

Supplementary Note 2: Stability of polyelectrolyte complexes against salt and urea.

Turbidity was used to investigate the stability of liquid and solid complexes as a function of both salt (NaCl) and denaturant (urea) concentration. While low concentrations of salts have been shown to enhance complex formation (Supplementary Fig. 5), higher concentrations of salt ultimately destabilize the phase transition. The concentration above which phase separation no longer occurs is known as the critical salt concentration. Supplementary Fig. 5 presents the turbidity data for both liquid coacervates and solid precipitates. As can be seen, the trends in turbidity are more clearly defined for the case of liquid coacervates. This is expected because the surface area of liquid droplets is a more easily defined and reproducible result than the surface area associated with scattering from a solid precipitate. However, in both cases we observe significant variations in the critical salt concentration as a function of chirality. A summary of these results is presented in Supplementary Table 2, along with data that has been scaled to correct for variations in the molecular weight of the polymers. Taking into account these molecular weight effects, the stability of liquid coacervates formed from two racemic polymers (*i.e.*, p(*D,L*)K+p(*D,L*)E) was significantly lower than for complexes formed from only a single racemic polymer. For solid precipitates we observe significantly higher critical salt concentrations for complexes formed from polymers of opposite chirality (*i.e.*, p*L*K+p*D*E and p*D*K+p*L*E), than from those with matching chirality (*i.e.*, p*L*K+p*L*E and p*D*K+p*D*E).

While the effects of salt on complexation correlate with electrostatic and entropic forces, urea acts directly on peptide hydrogen bonds. Urea appeared to have little effect on liquid coacervates. This result was expected because of the random coil structure of the polypeptides, and thus the minimal contributions of hydrogen bonds to the structure. However, the addition of sufficient quantities of urea to solid precipitates resulted in the melting of solid complexes to a liquid state that very closely resembles liquid coacervates (Supplementary Fig. 6). Interestingly, we observe the same trends in stability against dissolution in urea as were observed with respect to salt.

Supplementary Note 3: Chirality effects in micellar polyelectrolyte complexes.

By fitting the CD data it was shown that SCMs have 100% β-sheet structure as can be seen in Supplementary Figs. 7a and 7c. Conversely, micelles formed using a racemic charged polypeptide maintained the random coil structure of the uncomplexed polypeptides illustrated in Supplementary Fig. 7b.

Examination of the effects of 1 M urea on LCM and SCM samples enabled characterization of peptide secondary structure via CD. As suggested by the phase transition in the bulk from a solid precipitate with β-sheet character to a liquid, the micellar systems transitioned from a SCM, which displayed 100% β-sheet character, evidenced by the characteristic minimum at 215 nm (Supplementary Fig. 7c), to a structure with 99.8% random coil micelle, reminiscent of a LCM, and characterized by a maximum near 220 nm and a minimum below 205 nm (Supplementary Fig. 7d). These results suggest that while electrostatic interactions provide a base level of stability, the way in which polypeptide chirality affects the number and strength of hydrogen bonding interactions can be used to tune material properties. Thus, an analogous solid to liquid transition occurs in microphase-separated systems, as was observed in bulk homopolymer systems.

Supplementary References

1. Baiz, C. R., Reppert, M. & Tokmakoff, A. in *Ultrafast Infrared Vibrational Spectroscopy* (Fayer, M. D.) 361–404 (CRC Press, 2012).
2. Kramer, J. R. & Deming, T. J. General method for purification of α-amino acid- N-carboxyanhydrides using flash chromatography. *Biomacromolecules* **11,** 3668–3672 (2010).
3. Fandrich, M. & Dobson, C. M. The behaviour of polyamino acids reveals an inverse side chain effect in amyloid structure formation. *EMBO J.* **21,** 5682–5690 (2002).
4. Spruijt, E. *et al.* Structure and dynamics of polyelectrolyte complex coacervates studied by scattering of neutrons, X-rays, and light. *Macromolecules* **46,** 4596–4605 (2013).
5. Zandomeneghi, G., Krebs, M. R. H., McCammon, M. G. & Fändrich, M. FTIR reveals structural differences between native β-sheet proteins and amyloid fibrils. *Protein Sci.* **13,** 3314–3321 (2009).
6. Dzwolak, W., Ravindra, R., Nicolini, C., Jansen, R. & Winter, R. The diastereomeric assembly of polylysine is the low-volume pathway for preferential formation of β-sheet aggregates. *J. Am. Chem. Soc.* **126,** 3762–3768 (2004).
7. Miyazawa, T. & Blout, E. R. The infrared spectra of polypeptides in various conformations: amide I and II bands. *J. Am. Chem. Soc.* **83,** 712–719 (1961).
